# Increased Expression of Adherens Junction Components in Mouse Liver following Bile Duct Ligation

**DOI:** 10.3390/biom9100636

**Published:** 2019-10-22

**Authors:** Raf Van Campenhout, Sara Crespo Yanguas, Axelle Cooreman, Eva Gijbels, Kaat Leroy, Vânia Vilas-Boas, Nick Devoogdt, Serge Muyldermans, Bruno Cogliati, Mathieu Vinken

**Affiliations:** 1 Department of In Vitro Toxicology and Dermato-Cosmetology, Vrije Universiteit Brussel, Laarbeeklaan 103, B-1090 Brussels, Belgium; raf.van.campenhout@vub.be (R.V.C.); screspo@idibell.cat (S.C.Y.); axelle.cooreman@vub.be (A.C.); eva.anne.gijbels@vub.be (E.G.); kaat.leroy@vub.be (K.L.); vania.vilas.boas@vub.be (V.V.-B.); 2 In Vivo Cellular and Molecular Imaging Laboratory, Vrije Universiteit Brussel, Laarbeeklaan 103, B-1090 Brussels, Belgium; ndevoogd@vub.be; 3 Laboratory of Cellular and Molecular Immunology, Vrije Universiteit Brussel, Pleinlaan 2, B-1050 Brussels, Belgium; serge.muyldermans@vub.be; 4 Department of Pathology, School of Veterinary Medicine and Animal Science, University of São Paulo, 05508-270 São Paulo, Brazil; bcogliati@usp.br

**Keywords:** cadherin, catenin, liver, fibrosis, cholestasis

## Abstract

Adherens junctions, consisting of cadherins and catenins, are a group of cell-to-cell junctions that mediate mechanistic linkage between neighboring cells. By doing so, adherens junctions ensure direct intercellular contact and play an indispensable role in maintaining tissue architecture. Considering these critical functions, it is not surprising that adherens junctions are frequently involved in disease. In the present study, the effects of bile duct ligation—a surgical procedure to experimentally induce cholestatic and fibrotic liver pathology—on hepatic adherens junctions were investigated in mice. In essence, it was found that liver mRNA and protein levels of E-cadherin, β-catenin and γ-catenin drastically increase following bile duct ligation. These results could suggest a cytoprotective role for hepatic adherens junctions following bile duct ligation.

## 1. Introduction

Adherens junctions form bridges that connect the cytoskeleton of adjacent cells and hence are key structures in the maintenance of cellular architecture [1]. They are built up by transmembrane and cytosolic proteins. The former belong to the superfamily of cadherins, with E-cadherin being the most prominent one expressed by epithelial cells. At the cytosolic site, cadherins are linked to the actin cytoskeleton via catenins [1,2]. In liver, hepatocytes abundantly express β-catenin and γ-catenin [3,4]. Besides linking to E-cadherin and providing structural and mechanistic support, catenins also participate in intracellular signaling. Indeed, in the canonical Wnt pathway, β-catenin moves to the cell nucleus and recruits transcriptional coactivators, thereby regulating the expression of several genes involved in various aspects of the cellular life cycle [5,6,7,8,9,10].

Given the critical roles of adherens junctions and their constituents in cellular homeostasis, it is not surprising that these structures are frequently involved in disease. In this respect, β-catenin levels steeply increase in acetaminophen-induced acute liver failure in mice [11]. Likewise, hepatic β-catenin quantities become progressively elevated in diet-induced non-alcoholic steatohepatitis in mice, while E-cadherin is negatively affected [12]. High levels of β-catenin are also found in liver fibrosis patients [13]. In addition, E-cadherin expression is negatively affected in hepatocellular carcinoma, while β-catenin and γ-catenin levels are upregulated [14].

The present study was initiated in order to investigate the effects of bile duct ligation (BDL), a well-known surgical procedure that triggers cholestatic and fibrotic insults, on hepatic adherens junctions in mice. Focus is put on monitoring changes in the expression and cellular localization of E-cadherin, β-catenin and γ-catenin.

## 2. Materials and Methods

### 2.1. Animals and Treatment

Male C57BL/6 mice were obtained from Jackson Laboratories (USA). Animals were housed in the animal facility of the School of Veterinary Medicine and Animal Science of the University of São Paulo. Mice were kept in a room with ventilation (i.e., 16–18 air changes/hour), relative humidity (i.e., 45–65%), controlled temperature (i.e., 20–24 °C) and light/dark cycle 12:12 and were given water and balanced diet (NUVILAB-CR1, Nuvital Nutrientes LTDA, Brazil) ad libitum. This study has been approved by the Committee on Bioethics of FMVZ-USP (protocol number 9999100314) and all animals received humane care according to the criteria outlined in the “Guide for the Care and Use of Laboratory Animals”. The BDL or sham models were set up in 8-weeks to 12-weeks-old mice as described elsewhere [15,16]. Mice were sacrificed 20 days after BDL by exsanguination during sampling under isoflurane-induced anesthesia. Blood collected by cardiac puncture was drawn into a heparinized syringe and centrifuged for 10 min at 1503× *g*, and serum was stored at −20 °C. Livers were excised and fragments were fixed in 10% phosphate-buffered formalin or snap-frozen in liquid nitrogen with storage at −80 °C.

### 2.2. Analysis of Serum Biochemical Parameters

Serum levels of alanine aminotransferase (ALT; IU/L), aspartate aminotransferase (AST; IU/L), alkaline phosphatase (ALP; IU/L), conjugated and total bilirubin (mg/dL) were measured with an automated spectrophotometric Labmax 240 analyzer (Labtest Diagnostica, Lagoa Santa, Brazil) after appropriate dilution of samples.

### 2.3. Histological Examination of Liver Collagen Content

For microscopic evaluation, formalin-fixed liver fragments were embedded in paraffin and 5 µm sections were stained with Sirius red for blinded evaluation of the liver as previously described [17]. Morphometric analysis of Sirius red staining was performed in 10 randomly selected fields per section from the left lobe (10× objective) using a Nikon Eclipse Ti microscope (Nikon, Tokyo, Japan). Semi-quantitative analysis of the fibrosis area was performed with ImageJ (National Institutes of Health, Bethesda, MD, USA) [18] and calculated by the formula [area of fibrosis (%) = total fibrosis area/total area of the liver tissue.

### 2.4. Reverse Transcription Quantitative Real-Time Polymerase Chain Reaction Analysis

Reverse transcription quantitative real-time polymerase chain reaction (RT-qPCR) analysis of liver tissue was assayed as explained elsewhere [19]. The Taqman probes and primers specific for the target and reference genes (Thermo Fisher Scientific, Waltham, MA, USA) are presented in Table 1. Relative alterations (fold change) in RNA levels were calculated according to the 2^(−ΔΔCq)^ formula [20].

### 2.5. Immunoblot Analysis

Immunoblot analysis of liver tissue was performed as previously described [19] with slight modifications. After electrophoresis and blotting, blocking of the nitrocellulose membranes was performed with 5% non-fatty milk in Tris-buffered saline solution containing 0.1% Tween 20. Membranes were incubated overnight at 4 °C with primary antibody directed against E-cadherin, β-catenin, γ-catenin (BD Biosciences, San Jose, CA, USA) and adenomatous polyposis coli (APC) (Santra Cruz, Dallas, TX, USA) followed by incubation for 1 h at room temperature with horseradish peroxidase-conjugated secondary antibody (Dako, Santa Clara, CA, USA). Primary antibodies used for immunoblotting are presented in Table 2. Detection of the proteins was carried out by means of enhanced chemiluminescence. For semi-quantification purposes, a well-known normalization method based on total protein loading was used to overcome the drawbacks associated with the use of housekeeping proteins [21,22,23,24]. E-cadherin, β-catenin, γ-catenin and truncated APC signals BDL-treated mice were normalized against total protein loading and expressed as relative alterations compared to sham-operated animals.

### 2.6. Immunohistochemistry Analysis

Flash frozen liver tissue samples were embedded in Tissue Freezing Medium^®^ (Leica, Wetzlar, Germany). Then, 5 µm liver sections were fixed in methanol for 15 min at −20 °C and blocked in blocking buffer based on 5% *w/v* donkey serum (Jackson Immunoresearch Inc., West Grove, PA, USA) during 1 h. Subsequently, liver sections were incubated with primary antibodies directed against E-cadherin, β-catenin and γ-catenin (Abcam, Cambridge, UK; Cell Signaling Technolgy, Danvers, MA, USA) in blocking buffer based on 5% *w/v* donkey serum (Jackson Immunoresearch Inc., West Grove, PA, USA) overnight at 4 °C. Primary antibodies used for immunohistochemistry analysis are presented in Table 2. After extensive rinsing with phosphate-buffered saline supplemented with 0.5% Tween-20, samples were incubated with polyclonal goat anti-rabbit Alexa Fluor^®^ 488-conjugated secondary antibody (Jackson ImmunoResearch Inc., West Grove, PA, USA) in blocking buffer based on 5% *w/v* donkey serum (Jackson ImmunoResearch Inc., West Grove, PA, USA). Thereafter, Vectashield with 4′,6-diamidino-2-phenylindol (Vector laboratories, Burlingame, CA, USA) was used to stain nuclei and as mounting medium. Detection was performed using a fluorescence microscope Nikon Eclipse Ti (Nikon, Tokyo, Japan).

### 2.7. Statistical Analysis

All data were expressed as mean with data range (i.e., minimum to maximum). Results were statistically processed by 2-tailed unpaired student t-tests and Welch’s correction or Mann–Whitney tests depending on the distribution (i.e., D’Agostino–Pearson normality test for large number of biological repeats (*n*) or Shapiro–Wilk normality test for low *n*) using GraphPad Prism7 software (GraphPad Software Inc., San Diego, CA, USA), with probability (*p*) values of less than or equal to 0.05 considered as significant.

## 3. Results

### 3.1. Characterization of the Bile Duct Ligation Model

**Figure 1:**
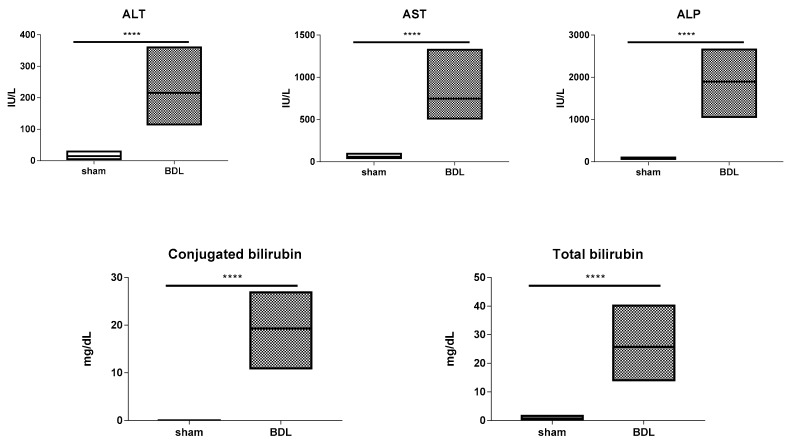
Analysis of biochemical parameters following bile duct ligation (BDL) in mice. Mice (*n* = 12–16) were subjected to BDL for 20 days. Serum levels of alanine aminotransferase (ALT), aspartate aminotransferase (AST), alkaline phosphatase (ALP), conjugated and total bilirubin were determined. Results were analyzed by 2-tailed unpaired student t-tests and Welch’s correction or Mann–Whitney tests. Data were expressed as means with data range (minimum to maximum) (**** *p* ≤ 0.0001).

**Figure 2:**
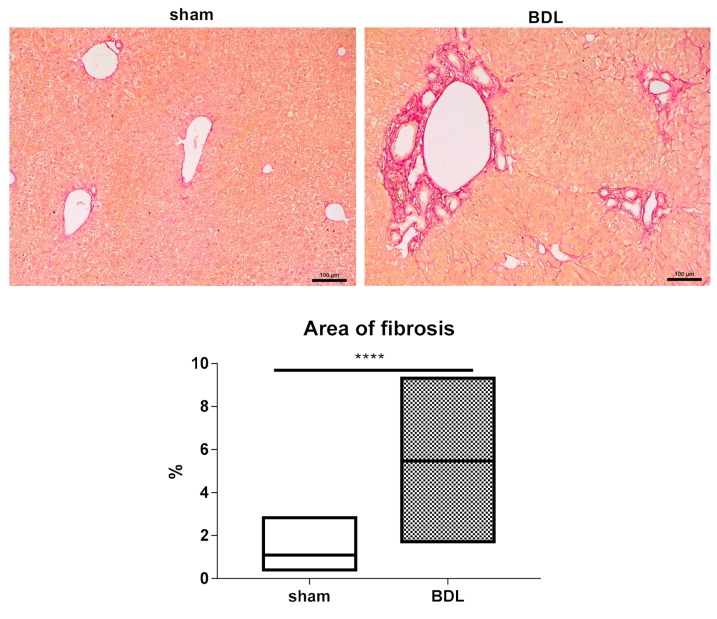
Morphometric analysis of liver collagen following bile duct ligation (BDL) in mice. Mice (*n* = 12-15) were subjected to BDL for 20 days. Collagen morphometric analysis was performed by quantification of the area of collagen fibers stained by Sirius red. Results were analyzed by Mann–Whitney test. Data were expressed as means with data range (minimum to maximum) (**** *p* ≤ 0.0001). Scale bar represents 100 µm.

Ligation of the extrahepatic bile duct in the BDL model causes cholestasis, as bile accumulates in the intrahepatic bile ducts to induce backpressure and shear stress, in turn leading to injury and fibrosis [25,26]. This complies with the results of the present study, showing an increase (*p* ≤ 0.0001) in serum levels of the general liver injury markers ALT and AST following 20 days of BDL. This coincided with elevated (*p* ≤ 0.0001) quantities of the cholestatic indicators ALP, conjugated and total bilirubin (Figure 1). Furthermore, morphometric analysis following Sirius red staining of hepatic collagen showed a significantly higher (*p* ≤ 0.0001) normalized collagen area ratio in liver tissue of BDL-subjected mice compared to sham-operated counterparts (Figure 2).

### 3.2. Effects of Bile Duct Ligation on Hepatic Adherens Junctions

**Figure 3:**
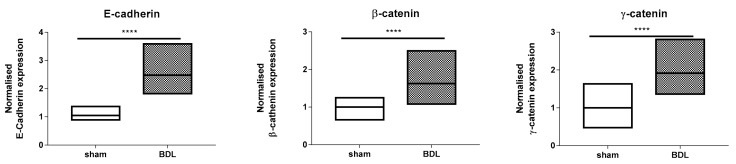
Analysis of adherens junction gene expression following bile duct ligation (BDL) in mice. Mice (*n* = 10–17) were subjected to BDL for 20 days. RNA was extracted from the liver samples and subjected to RT-qPCR analysis of E-cadherin, β-catenin and γ-catenin. Fold changes in RNA levels were calculated, whereby the average expression of sham-operated animals was set to 1. Results were analyzed by 2-tailed unpaired student t-tests and Welch’s correction. Data were expressed as means with data range (minimum to maximum) (**** *p* ≤ 0.0001).

**Figure 4:**
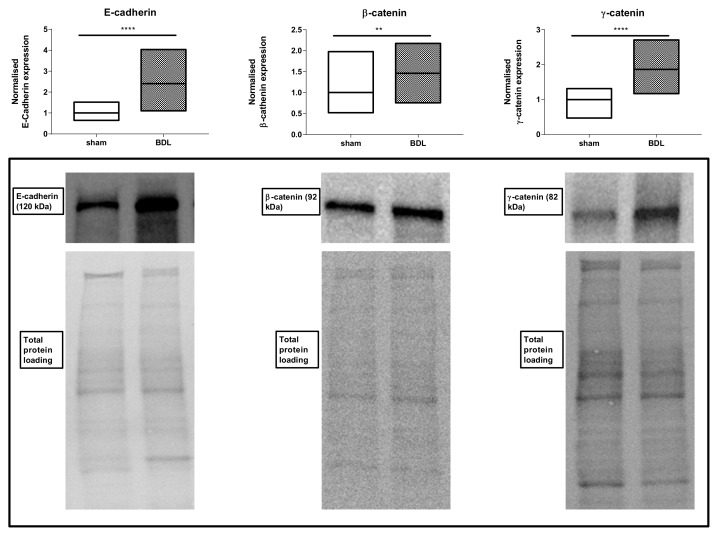
Analysis of adherens junction protein expression following bile duct ligation (BDL) in mice. Mice (*n* = 12–18) were subjected to BDL for 20 days. Hepatic protein levels of E-cadherin, β-catenin and γ-catenin were assessed by immunoblot analysis, normalized against the total protein content and expressed as relative alteration compared to sham-operated animals. Results were analyzed by 2-tailed unpaired student t-tests and Welch’s correction. Data were expressed as means with data range (minimum to maximum) (** *p* ≤ 0.01; **** *p* ≤ 0.0001).

**Figure 5:**
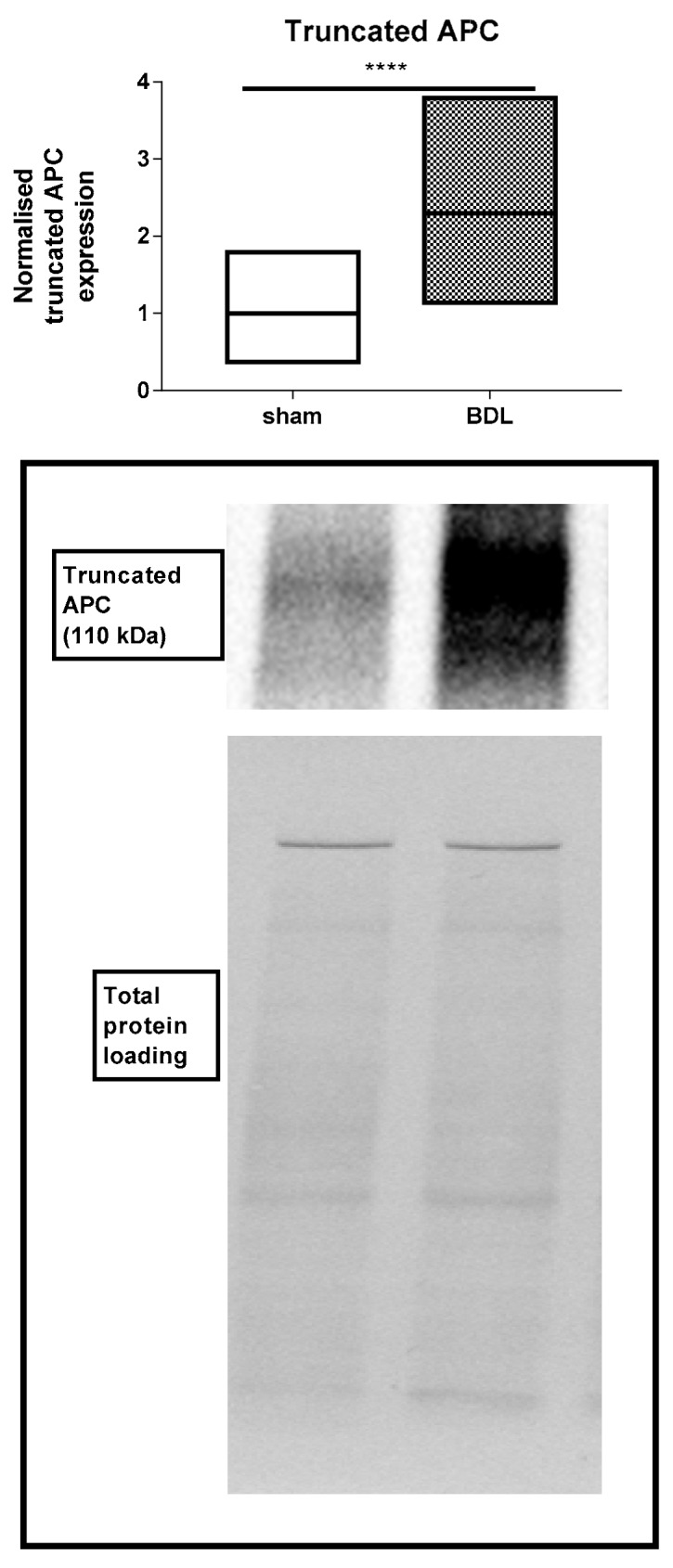
Analysis of truncated adenomatous polyposis coli (APC) protein expression following bile duct ligation (BDL) in mice. Mice (*n* = 11–12) were subjected to BDL for 20 days. Hepatic protein levels of APC were assessed by immunoblot analysis, normalized against the total protein content and expressed as relative alteration compared to sham-operated animals. Results were analyzed by 2-tailed unpaired student t-tests and Welch’s correction. Data were expressed as means with data range (minimum to maximum) (**** *p* ≤ 0.0001).

**Figure 6:**
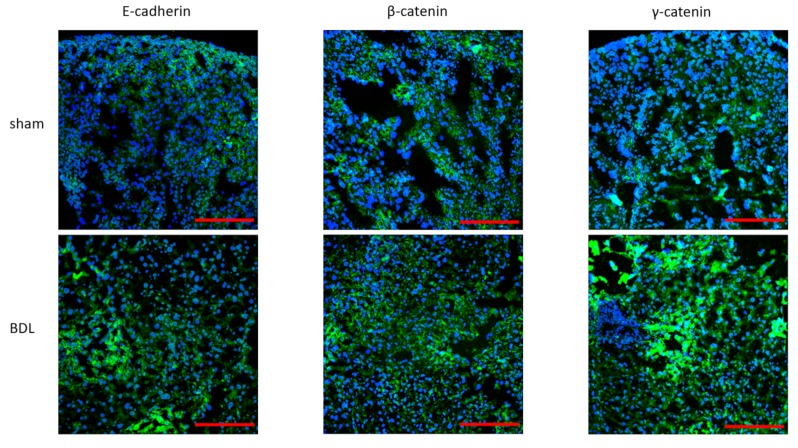
Analysis of adherens junction cellular localization following bile duct ligation (BDL) in mice. Mice (*n* = 12–18) were subjected to BDL for 20 days. Liver sections were prepared and subjected to immunohistochemistry analysis of E-cadherin, β-catenin and γ-catenin (green color) with nuclear 4′,6-diamidino-2-phenylindol counterstaining (blue color). Scale bar represents 200 µm.

Following characterization of the liver disease model as such, effects of BDL applied for 20 days on hepatic adherens junctions was investigated. RT-qPCR analysis showed a steep increase (*p* ≤ 0.0001) in mRNA quantities of liver E-cadherin, β-catenin and γ-catenin (Figure 3). These transcriptional changes were fully translated at the protein level as evidenced by upregulated amounts of E-cadherin (*p* ≤ 0.0001), β-catenin (*p* ≤ 0.01) and γ-catenin (*p* ≤ 0.0001) (Figure 4). Besides, liver protein levels of truncated APC also significantly increased following BDL (*p* ≤ 0.0001) (Figure 5). The enhanced presence in liver of the 3 adherens junction components upon BDL was also confirmed by immunohistochemistry analysis (Figure 6).

## 4. Discussion

As such, BDL is known to differentially affect cell-to-cell junctions in liver, including tight junctions [27], desmosomes [28] and gap junctions [29]. The present study intended to extend this knowledge by studying the impact of this experimental surgical procedure on adherens junctions, being critical mediators of hepatic homeostasis that secure mechanistic anchorage and that participate in cellular signaling [30,31]. It was found that protein moieties of E-cadherin, β-catenin and γ-catenin in mouse liver drastically increase upon BDL. This was mirrored by similar changes in mRNA quantities, suggesting that the general increase in production of adherens junction components triggered by BDL relies on transcriptional activity. BDL damages liver tissue, which is composed of epithelial and mesenchymal cells followed by activation of repair mechanisms to reconstruct normal hepatic structure and function [32,33]. In fact, BDL-induced liver injury might be associated with type 2 epithelial-to-mesenchymal transition (EMT) [32,33,34]. The latter is a fibrosis-associated process, which has been shown to contribute to BDL-induced damage in mice, whereby epithelial cells undergo metamorphosis. Epithelial cells hereby acquire migratory and mesenchymal hallmarks as evidenced by expression of β-catenin and evolve to collagen producing-fibroblasts [35,36,37]. Thus, these transition and migration events contribute to the accumulation of activated fibroblasts and the subsequent development of liver fibrosis [35,38]. EMT, which is modulated at the transcriptional level, is furthermore accompanied by a unique manner of cell conversion [39]. During the intermediate stage of type 2 EMT, involved epithelial cells do not only gain mesenchymal markers, like β-catenin, but also possess an epithelial nature by securing the expression of epithelial-specific E-cadherin [35,40,41]. Expression of epithelial and mesenchymal markers is therefore used to recognize cells undergoing EMT [32]. Hence, promoted type 2 EMT activity could possibly underlie the increased expression of adherens junction components, in casu E-cadherin and β-catenin, in mouse liver following BDL. Additionally, co-expression of other epithelial and mesenchymal markers has already been demonstrated in EMT-involved cells, which contributes to portal tract fibrogenesis during human chronic liver disease [42]. Cells undergoing the complete EMT-process lose eventually their epithelial markers and acquire a full fibroblastic phenotype [35,41].

On the other hand, the BDL-associated alteration of hepatic adherens junction components might equally affect cellular signaling in hepatocytes. As observed in the present study, BDL treatment elevates total β-catenin protein levels in liver. This could lead to activation of the Wnt pathway and enhanced expression of target genes, in turn promoting proliferation [7]. This also holds true for γ-catenin, which is equally involved in the Wnt pathway and that participates in cellular signaling events to reconstruct normal hepatic structure and function [43,44,45].

Additionally, liver APC protein expression levels were investigated. Immunoblot analysis showed that the BDL model causes an increase in truncated APC. APC mediates intercellular adhesion by targeting β-catenin and γ-catenin for degradation. Therefore, APC proteins are regulators of catenin levels and are described as components of the canonical Wnt pathway [46,47]. In this way, APC influences cellular signaling in hepatocytes. Moreover, protein expression of APC and β-catenin is also involved in liver zonation. Elevated β-catenin and APC expression is described in perivenous and periportal regions, respectively. Consequently, an increase of APC and the link between APC proteins, Wnt signaling and catenin levels may be correlated with hepatic repair and zonation processes in mouse liver following BDL [48,49].

The relevance of increased expression of adherens junction building stones upon BDL remains to be elucidated. Enhanced hepatic E-cadherin production following BDL in mice was also observed by other groups [50]. Furthermore, β-catenin signaling has been demonstrated to regulate hepatobiliary repair in mice subjected to BDL [48]. Additionally, when subjected to BDL, γ-catenin knock-out mice are more prone to fibrotic and cholestatic injury compared to wild-type counterparts [28]. Similarly, dual β-catenin and γ-catenin loss in murine liver causes progressive intrahepatic cholestasis and fibrosis [51]. These results are in line with the outcome of the present study and could suggest a cytoprotective role for adherens junction components following BDL. Such a role could be the promotion of regenerative capacity in order to compensate for the loss of cells during the actual insult [52,53,54].

## Figures and Tables

**Table 1:** Primers and probes for RT-qPCR analysis of target and candidate reference genes. Assay identification (ID), accession number, assay location, amplicon length and exon boundary of target and candidate reference genes are presented (Actb, β-actin; B2m, β-2-microglobulin; Cdh1, E-cadherin; Ctnnb1, β-catenin; Gapdh, glyceraldehyde 3-phosphate dehydrogenase; Hmbs, hydroxymethylbilane synthase; Jup, γ-catenin; Ubc, ubiquitin C).

Gene Symbol	Assay ID	Accession Number	Assay Location	Amplicon Size (Base Pairs)	Exon Boundary
*Actb*	*Mm00607939_s1*	*NM_007393.3*	*1233*	*115*	*6-6*
*B2m*	*Mm00437762_m1*	*NM_009735.3*	*111*	*77*	*1-2*
*Cdh1*	*Mm01247357_m1*	*NM_009864.2*	*1452*	*71*	*9-10*
*Ctnnb1*	*Mm00483039_m1*	*NM_007614.3*	*2366*	*77*	*13-14*
*Gapdh*	*Mm99999915_g1*	*NM_008084.3*	*265*	*107*	*2-3*
*Hmbs*	*Mm01143545_m1*	*NM_013551.2*	*473*	*81*	*6-7*
*Jup*	*Mm00550256_m1*	*NM_034723.1*	*1657*	*66*	*8-9*
*Ubc*	*Mm02525934_g1*	*NM_019639.4*	*370*	*176*	*2-2*

**Table 2:** Primary antibodies used for immunoblotting and immunohistochemistry analysis. (IB, immunoblotting; IHC, immunohistochemistry).

Antigen	Supplier	Cat. No.	Species	Type	Dilution
**E-cadherin**	*BD Biosciences*	*610181*	*Mouse*	*Monoclonal*	*1/5000 (IB)*
	*Cell Signaling Technology*	*CST 3195S*	*Rabbit*	*Monoclonal*	*1/200 (IHC)*
**β-catenin**	*BD Biosciences*	*610153*	*Mouse*	*Monoclonal*	*1/1000 (IB)*
	*Abcam*	*Ab2365*	*Rabbit*	*Polyclonal*	*1/200 (IHC)*
**γ-catenin**	*BD Biosciences*	*610253*	*Mouse*	*Monoclonal*	*1/1000 (IB)*
	*Cell Signaling Technology*	*CST 2309S*	*Rabbit*	*Polyclonal*	*1/200 (IHC)*
**APC**	*Santa Cruz*	*sc-9998*	*Mouse*	*Monoclonal*	*1/1000 (IB)*
